# The valence-dominance model applies to body perception

**DOI:** 10.1098/rsos.220594

**Published:** 2022-09-07

**Authors:** Eva Tzschaschel, Kevin R. Brooks, Ian D. Stephen

**Affiliations:** ^1^ School of Psychological Sciences, Macquarie University, 4 First Walk, North Ryde, New South Wales 2109, Australia; ^2^ Perception in Action Research Centre, Macquarie University, Sydney, Australia; ^3^ Body Image and Ingestion Group, Macquarie University, Sydney, Australia; ^4^ School of Social Sciences, Nottingham Trent University, Nottingham, UK

**Keywords:** perception, social judgements, body perception, valence-dominance model

## Abstract

First impressions of a person, including social judgements, are often based on appearance. The widely accepted valence-dominance model of face perception (Oosterhof and Todorov 2008 *Proc. Natl Acad. Sci. USA* **105**, 11 087–11 092 (doi:10.1073/pnas.0805664105)) posits that social judgements of faces fall along two orthogonal dimensions: trustworthiness (valence) and dominance. The current study aimed to establish the principal components of social judgements based on the perception of bodies, hypothesizing that these would follow the same dimensions as face perception. Stimuli were black and white photographs showing bodies dressed in grey clothing, standing in their natural posture, in left profile. Raters (*N* = 237) judged the stimuli on the 14 traits used in Oosterhof and Todorov's original study (Oosterhof and Todorov 2008 *Proc. Natl Acad. Sci. USA* **105**, 11 087–11 092 (doi:10.1073/pnas.0805664105)). Data were analysed using principal component analysis (PCA), as in the original study, with an additional exploratory factor analysis (EFA) using oblique rotation. While PCA produced a third dimension in line with several replications of the original study, results from the EFA produced two dimensions, representing trustworthiness and dominance, providing support for the hypothesis that social perceptions of bodies can be summarized using the valence-dominance model. These two factors could represent universal perceptions we have about people. Future research could explore social judgements of humans based on other stimuli, such as voices or body odour, to evaluate whether the trustworthiness and dominance dimensions are consistent across modalities.

## 1. The valence-dominance model applies to body perception

People often form first impressions of others based on their physical appearance [1]. These impressions go beyond physical attractiveness and include social judgements of traits such as perceived intelligence, dominance and confidence [2–4]. Furthermore, there is some evidence that observers' social judgements are predictive of individuals’ actual personality traits; for example, positive correlations have been found between individuals' self-reported extraversion and perceived extraversion as rated by observers based on the images of their faces [5].

Oosterhof & Todorov [6] asked 55 participants in an initial study to freely associate traits to standardized face photographs from the Karolinska database. From this initial study 13 trait judgements were identified as trait dimensions. The authors then conducted seven follow-up studies where 327 raters judged between 20 and 300 computer-generated imagery (CGI) faces with neutral facial expressions on these 13 traits, plus dominance, which was included due to its importance in interpersonal perception. A principal component analysis (PCA) showed that social judgements of faces can be summarized by two orthogonal underlying dimensions—trustworthiness (valence) and dominance. Social judgements of a range of facial stimulus types have been found to follow the same underlying dimensions of valence and dominance, for example using photographs taken from various angles, under different lighting conditions and with various natural facial expressions [7], morphed photographs of children's and adults’ faces [8,9] and stylized, decomposed and partly obstructed photographs [10]. Furthermore, the model's robustness has also been tested across a wide range of cultures, finding that the model is supported in most world regions [11]. However, among statisticians and researchers, there have been discussions regarding the initial statistical analysis, i.e. PCA, that Oosterhof & Todorov [6] used. In some instances, scholars suggest an exploratory factor analysis (EFA), which has been described as more appropriate for identifying unknown dimensions [12]. Others recommend running both PCA and EFA on datasets to explore the differences [13]. A recent project conducted by Jones *et al*. [11] analysed the data from their studies using both PCA with orthogonal rotation and EFA with oblique rotation and found variation in the dimensions produced.

Further research is emerging, suggesting that the valence-dominance model may also apply to perception of computer-generated voice stimuli [14]. Research on body perception, meanwhile, is less extensive, and has typically focused on individual social perceptions, such as perceptions of attractiveness or health, in isolation [15,16]. Therefore, it is not known whether social perceptions of bodies may also be summarized along valence and dominance dimensions. However, in recent years, researchers have investigated whether perception of bodies is comparable to face perception [17,18]. For example, Hu *et al*. [18] presented three-dimensional computer-generated body shapes to observers who rated them on two traits from the Big Five personality scale. Their findings indicate that participants make inferences from various body shapes, such as valence from body size and agency from body shape (i.e. broad shoulders, rectangular shape). Morrison *et al*. [17] compared face perception and body perception, finding that PCA of face ratings resulted in components similar to Oosterhoff & Todorov's [6] valence-dominance model, while ratings of bodies resulted in a single underlying component, which they called the *body general component*. The body general component reflected both valence and dominance [17]. Although this suggests that the valence-dominance model may not apply to body perception, Morrison *et al*.'s [17] study differed from Oosterhof and Todorov's original study in that the images used were full colour photographs of young, nude volunteers (faces and primary genitals obstructed with black circles), featuring various body shapes, compared with Oosterhof & Todorov's [6] use of CGI faces and photographs of members of the general public during the initial phase of trait association. Further, Morrison *et al*. [17] also used PCA, which has been criticized for robustness by various scholars. The relative qualities of PCA and EFA have been summarized by Widaman [19], who recommends the use of EFA with oblique rotation as an alternative to PCA.

Here, we report the results of a study in which observers rated photographs of human bodies on Oosterhof and Todorov's 14 traits to test their model on body perception. We then use PCA as well as EFA to determine whether social perceptions of bodies can be summarized along the valence and dominance dimensions. We hypothesized that two dimensions would be produced: trustworthiness (valence) and dominance.

## 2. Method

This research was approved by the Macquarie University Human Research Ethics Committee (MQ HREC). All participants were naive to the hypothesis and gave informed consent in writing before participating for course credit. We excluded 15 participants who did not finish the study and 14 participants for providing the same stimulus rating on more than 80% of trials. The participants were Macquarie University undergraduates (*N* = 237: 109 males, 128 females) from various ethnic backgrounds, aged between 18 and 66 (*M* = 24.76, s.d. = 9.02).

### 2.1. Measures

**Figure 1.**
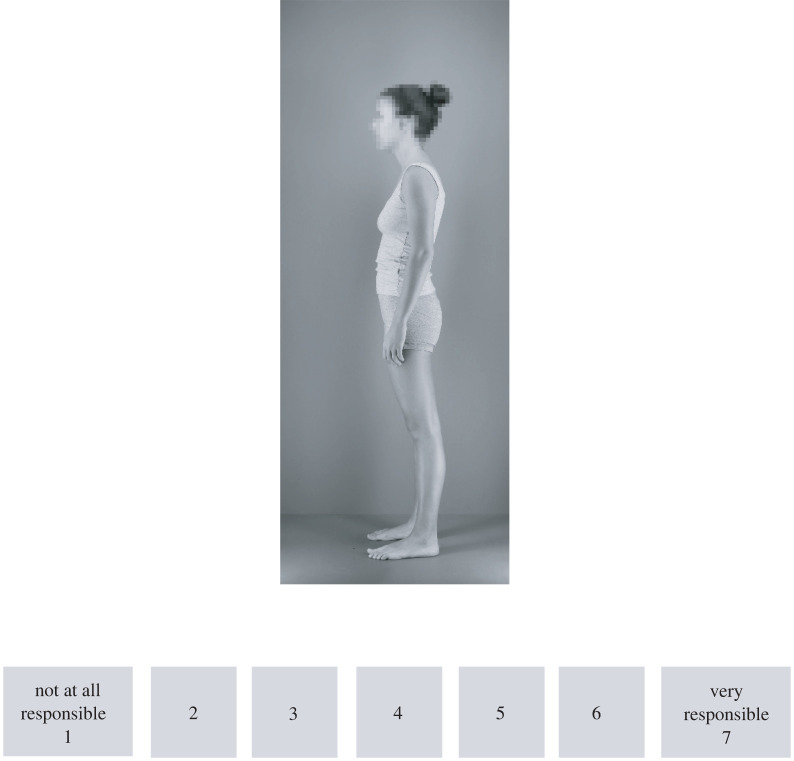
Example rating stimulus.

Participants were presented with a series of male (58) and female (63) bodies using Qualtrics software (Qualtrics, Provo). Each body photograph was shown in left profile view standing in natural posture (figure 1). The stimuli were created by photographing 121 Caucasian volunteers, who gave informed consent in writing for their photographs to be used in further studies. Age ranged from 18 to 30 for males (*M* = 21.9 years, s.d. = 3.7), from 17 to 33 for females (*M* = 20.5 years, s.d. = 4.7), whereas height ranged from 166 to 199 cm for males (*M* = 182.1 cm, s.d. = 8), from 153 to 183 cm for females (*M* = 167 cm, s.d. = 6.9) and BMI ranged from 17.5 to 31.7 for males (*M* = 24 BMI, s.d. = 3.3), from 17.9 to 30.1 for females (*M* = 22 BMI, s.d. = 2.7).

Photographs were taken in a photobooth (117 × 90 × 210 cm), which was painted with Munsell N5 neutral grey paint. Fifteen Phillips T12/D65 daylight simulating fluorescent tubes were used to illuminate the booth in an otherwise darkened room. Flicker was reduced through high frequency fixtures and even light distribution was achieved by Perspex diffusers. A Canon EOS 70D DSLR camera with an 18–55 mm lens (focal length held constant for all images), was mounted on a tripod 1 m above the floor and 3 m away from the booth. Camera settings were the same for all images at 1/50 s exposure time, a lens aperture of F/5.6, white balance set at 6500 K and an ISO speed rating of 200. Photographs were presented in greyscale to reduce the potential confounding factor of skin colour and the faces were pixelated using the pixelate function of Adobe Photoshop CC 2017 (Windows version) with a pixelate level of 35 × 35 to ensure that observers based their ratings on the bodies and not the faces. Original jpg images are 1794 × 4494 pixels with each enlarged pixel produced by averaging an area comprising 35 × 35 of the original pixels. Each enlarged pixel is, therefore, approximately 2% of the width of the image.

Bodies were rated on the same 14 traits as in the original Oosterhof & Todorov [6] study (sociable, dominant, trustworthy, responsible, caring, [un]happy, emotionally stable, aggressive, intelligent, mean, weird, self-esteem, confident and attractive). The stimuli were presented in random order in 14 blocks (one block per trait). Each block was introduced with instructions, for example: ‘For this next block, please rate how RESPONSIBLE each person looks.’ Answers were given via a Likert type scale from 1 to 7, labelled as 1 ‘Not at all responsible’ to 7 ‘Very responsible’ (figure 1).

### 2.2. Procedure

After signing up for the study, participants received a Qualtrics survey link to be accessed within 30 min from their own computers, tablets or mobile phones. Participants completed an informed consent page, answered basic demographic questions (age, sex and ethnicity), and were then directed to the survey. Each participant was presented with all stimuli and asked to make two judgements (randomly selected from the 14 traits) of each body to avoid fatigue.

### 2.3. Statistical methods

The statistical analysis used by Oosterhof & Todorov [6] was later criticized by scholars claiming that PCA is not suitable for understanding the dimensional structure of social judgement data, because it does not assume the presence of unknown dimensions or latent factors explaining the responses [18]. Instead, EFA is preferred, since it makes the assumption of unknown dimensions and thus produces more stable solutions [11,18]. Further, an oblique rotation is advised, since there is no *a priori* theoretical reason to assume factors will be orthogonal. Hence, we decided to analyse our data twice: firstly, using PCA with orthogonal rotation to replicate Oosterhof & Todorov's [6] model directly, and secondly using EFA with oblique rotation to address the aforementioned criticisms. Data analysis was performed in JASP, v. 0.92 and RStudio, v. 1.2.5019.

Further, we related the extracted components to stimulus variables such as sex, age, height, BMI, muscularity and body fat percentage by using correlations. These analyses as well as the descriptive statistics for the stimuli were completed in JASP, v. 0.16.2.0.

## 3. Results

### 3.1. Replication of Oosterhof and Todorov's principal component analysis

PCA using Kaiser's criterion to retain components (eigenvalues greater than 1) yielded three principal components. The first component (PC1) accounted for 51.6% of the variance, whereas the second (PC2) and third (PC3) components explained 26.1% and 7.3% of the variance, respectively (table 1).

**Table 1.** Structure matrix of the principal component analysis. Loadings smaller than 0.4 are not shown.

	PC1	PC2	PC3
sociable	0.875		
dominant	0.720	0.617	
trustworthy	0.552	−0.660	
responsible	0.785	−0.434	
caring	0.519	−0.580	
unhappy	0.841		0.446
emotionally stable	0.947		
aggressive		0.907	
intelligent	0.672	−0.580	
mean		0.787	−0.461
weird	−0.615		0.541
self-esteem	0.885		
confidence	0.863		
attractive	0.847		

We were aiming to establish whether there were two main dimensions, valence and dominance, in our study like the original model [6].

### 3.2. Results of the exploratory factor analysis

**Figure 2.**
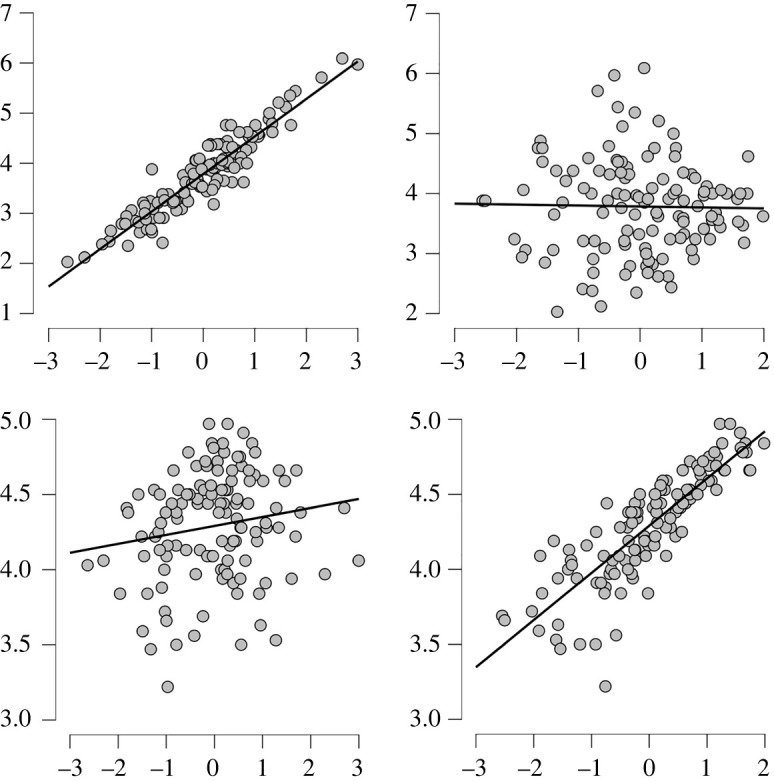
Scatterplots showing relationships between ‘dominant’ and ‘trustworthy’ and factors 1 and 2.

Following Jones *et al*. [11], we performed EFA with oblique rotation. Parallel analysis indicated the retention of two factors [19]. A Kaiser–Meyer–Olkin test showed that sampling was adequate for factor analysis (measure of sampling adequacy (MSA) = 0.87). Factor 1 explained 42.3% of the variance, and factor 2 explained 32% of the variance. The traits dominant, sociable, [un]happy, emotionally stable, aggressive, mean, self-esteem and confidence loaded onto factor 1. Trustworthy, responsible, caring and intelligent loaded onto factor 2 (table 2). [Un]happy, emotionally stable and attractive loaded onto both factors. Pearson's correlations (figure 2) show that ratings of dominance and trustworthiness do not significantly correlate with each other *r*_121_ = −0.057, *p* = 0.540. Dominance correlates strongly with factor 2, *r*_121_ = 0.936, *p* = < 0.001, but not with factor 1, *r*_121_ = 0.147, *p* = 0.108, whereas trustworthiness correlates strongly with factor 1, *r*_121_ = 0.770, *p* = <0.001 and not with factor 2, *r*_121_ = 0.028, *p* = 0.765. Therefore, EFA did produce two underlying factors representing dominance and trustworthiness, similar to Oosterhof and Todorov's valence-dominance findings for faces.

**Table 2.** Structure matrix for the exploratory factor analysis. Loadings smaller than 0.4 are not shown.

	factor 1	factor 2
sociable	0.892	
dominant	0.927	
trustworthy		0.828
responsible	0.465	0.826
caring		0.721
[un]happy	0.774	0.423
emotionally stable	0.823	0.609
aggressive	0.663	−0.546
intelligent		0.861
mean	0.422	−0.493
weird		−0.631
self-esteem	0.942	
confidence	0.934	
attractive	0.646	0.641

Exploratory analyses found that factor 1 (dominance) is significantly associated with male sex (*t*_119_ = −4.601, *p* < 0.001, *d* = −0.84), with male stimuli (*M* = 0.40, s.d. = 0.99) scoring higher than female stimuli (*M* = −0.37, s.d. = 0.85), as well as taller height (*r*_121_ = 0.36, *p* < 0.001), higher BMI (*r*_120_ = 0.25, *p* = 0.006), more muscle mass (*r*_121_ = 0.45, *p* < 0.001) and older age (*r*_121_ = 0.28, *p* = 0.002), consistent with the interpretation of this factor as representing dominance. The correlation of Factor 1 (*r*_121_ = −0.17, *p* = 0.065) with body fat was not significant. Factor 2 (valence) was associated with female sex (*t*_119_ = 7.30, *p* < 0.001, *d* = 1.31) with female stimuli (*M* = 0.52, s.d. = 0.84) scoring higher than male stimuli (*M* = −0.56, s.d. = 0.79), as well as shorter height (*r*_121_ = −0.33, *p* < 0.001), lower BMI (*r*_121_ = −0.49, *p* < 0.001), less muscle mass (*r*_121_ = −0.53, *p* < 0.001) and younger age (*r*_121_ = −0.27, *p* = 0.003), consistent with the interpretation of this factor as representing valence. The correlation of factor 2 (*r*_121_ = −0.15, *p* = 0.093) with body fat was also not significant.

## 4. Discussion

It was hypothesized that perception of human bodies would follow Oosterhof and Todorov's valence-dominance model, as does perception of human faces. The hypothesis was supported by the EFA but was not supported in the PCA.

We firstly applied the PCA technique of Oosterhof & Todorov [6] with similar results, but our data showed a third component with an eigenvalue just over 1, whereas Oosterhof and Todorov’s analysis produced a third factor with an eigenvalue just below 1. Oosterhof & Todorov [6] applied Kaiser's criterion in determining how many components to retain and interpret, leading them to discard the third component which had an eigenvalue less than 1. However, since several replication attempts have found this third component to have an eigenvalue greater than 1 in faces and now in bodies, it may be that this third component warrants interpretation. However, as Jones *et al*. [11] conclude, since this third component explains a relatively small amount of variance (10% or less in each world region) it may not be of theoretical interest. Following this logic, the third component identified in our data may also be of little theoretical interest since it explains only 7.3% of variance in body ratings.

We also found differences between the loadings of some of the trait dimensions in our study versus Oosterhof & Todorov's [6] PCA findings. Most notably, whereas Oosterhof & Todorov [6] found two distinct components—trustworthiness and dominance—for faces, our data showed that dominance loaded positively onto both components for bodies. However, because we assume that PCA has its drawbacks for a dataset with unknown dimensions, these results derived from PCA may have to be viewed with caution.

Oosterhoff and Todorov's statistical approach has recently been criticized on three grounds [19]. First, PCA does not assume that there are unknown dimensions causing the trait ratings of the stimuli, whereas the theory advanced by Oosterhof & Todorov [6] suggests that such latent factors do exist. EFA does make this assumption and hence has the advantage of providing a more stable representation of the latent structure of the data [19]. Second, Oosterhof & Todorov [6] assume that the dimensions of face perception are orthogonal, while theory does not dictate that they should be. The oblique rotation technique allows the identification of factors that correlate with one another. Third, Horn's parallel analysis is thought to be a more accurate method for determining the number of factors to retain than Kaiser's criterion [20]. Applying an EFA to our body perception data in light of these concerns produced two distinct factors—trustworthiness and dominance. While the order of the factors was reversed compared with Oosterhof & Todorov's [6] original findings, this represents a successful replication of their model in bodies.

Exploratory analyses of the associations between the two factors and stimulus characteristics (sex, age, height, muscularity, body fat and BMI) revealed that factor 1 (dominance) is correlated with male sex, more mature age, taller height, higher muscle mass and higher BMI but not with body fat. These results are consistent with previous literature with regards to perception of taller men, who are often seen as more dominant, of higher social status and higher earning potential [21,22] and that more masculine and older men are perceived as more dominant [23]. The findings related to body compositions could support previous research that more muscular but not leaner body composition is associated with increased perceived masculinity [24]. Factor 2 (trustworthiness) was related to youthfulness, female sex and smaller body sizes with less muscularity. This is potentially in line with previous studies showing that less threatening and more attractive faces are perceived as more trustworthy [25]. Future research should seek to identify the specific body traits that predict social perceptions of bodies along the valence-dominance axes.

Our study used greyscale photographs of real bodies, showing persons viewed in their left profile wearing grey clothing (shorts and singlets) instead of colour computer-generated stimuli. Faces were pixelated to reduce the possibility that judgements would instead be based on properties of the subjects' faces. However, as some features such as hair and facial characteristics such as the jawline were still partially visible, this could have still influenced the ratings of the stimuli. Nevertheless, the use of natural images could also be an advantage when it comes to body features, as there was natural variation of body size, shape, height and posture. According to the results of our study using EFA, the valence-dominance model holds with greyscale photographs of real-life bodies.

Contrary to our results, Morrison *et al*. [17] observed only one principal component for body perception that was correlated with traits for both valence and dominance. However, they only performed PCA in their study, and not EFA. Their stimuli were also photographs of real people, as in our study, but were photographed nude in frontal view (male and female chests exposed, but faces and genitals blocked out), while ours were clothed and photographed in profile view. Additionally, our stimuli had been converted into black and white photographs to reduce the potentially confounding effects of skin tone which had not been done with Morrison *et al*.'s [17] stimuli, although it is still possible that some skin tone information may be perceived in black and white photographs. Finally, while the faces of Morrison *et al*.'s [17] stimuli were fully obscured with a solid colour mask, the pixilation technique used in the current study did not fully obscure all facial information, which may, therefore, have played some role in the perception of the stimuli. Considering these variations, inconsistent results could be expected. This suggests the merit of further research into perception of bodies and faces with regards to social judgement.

In conclusion, our study aimed to extend the work of Oosterhof and Todorov by assessing whether their valence-dominance model of face perception applies to body perception. Our results using EFA provide evidence that body perception also follows the valence-dominance model. We propose that the factors of valence and dominance may underlie social judgements of humans in general, rather than applying only to judgements made from viewing faces alone. While evidence is provided here of the importance of these dimensions for forming first impressions when viewing bodies, it could be that, for example, body odour or voices (already under preliminary investigation) are also perceived according to valence and dominance. It would be worthwhile to continue exploring social judgements of humans based on other stimuli to assess whether the same dimensions continue to emerge.

## Data Availability

Data available from the Dryad Digital Repository: https://doi.org/10.5061/dryad.8931zcrth [26].
